# Hydrophobicity of Antifungal β-Peptides Is Associated with Their Cytotoxic Effect on *In Vitro* Human Colon Caco-2 and Liver HepG2 Cells

**DOI:** 10.1371/journal.pone.0149271

**Published:** 2016-03-18

**Authors:** Camilo Mora-Navarro, Janet Méndez-Vega, Jean Caraballo-León, Myung-ryul Lee, Sean Palecek, Madeline Torres-Lugo, Patricia Ortiz-Bermúdez

**Affiliations:** 1 Department of Chemical Engineering, University of Puerto Rico, Mayagüez Campus, Mayagüez, Puerto Rico; 2 Department of Chemical and Biological Engineering, University of Wisconsin-Madison, Madison, Wisconsin, United States of America; Louisiana State University, UNITED STATES

## Abstract

The widespread distribution of fungal infections, with their high morbidity and mortality rate, is a global public health problem. The increase in the population of immunocompromised patients combined with the selectivity of currents treatments and the emergence of drug-resistant fungal strains are among the most imperative reasons to develop novel antifungal formulations. Antimicrobial β-peptides are peptidomimetics of natural antimicrobial peptides (AMPs), which have been proposed as developmental platforms to enhance the AMPs selectivity and biostability. Their tunability allows the design of sequences with remarkable activity against a wide spectrum of microorganisms such as the human pathogenic *Candida spp*., both in planktonic and biofilm morphology. However, the β-peptide’s effect on surrounding host cells remains greatly understudied. Assessments have mainly relied on the extent of hemolysis that a candidate peptide is able to cause. This work investigated the *in vitro* cytotoxicity of various β-peptides in the Caco-2 and HepG2 mammalian cell lines. Results indicated that the cytotoxic effect of the β-peptides was influenced by cell type and was also correlated to structural features of the peptide such as hydrophobicity. We found that the selectivity of the most hydrophobic β-peptide was 2–3 times higher than that of the least hydrophobic one, for both cell types according to the selectivity index parameter (IC50/MIC). The IC50 of Caco-2 and HepG2 increased with hydrophobicity, which indicates the importance of testing putative therapeutics on different cell types. We report evidence of peptide-cell membrane interactions in Caco-2 and HepG2 using a widely studied β-peptide against *C*. *albicans*.

## 1. Introduction

Fungal infections are a worldwide human health threat due to their high morbidity and mortality rates [1]. This, along with the wide distribution of immunocompromised patients, the side effects of current antifungal treatments, and the emergence of drug resistant organisms represent the driving force behind antifungal drug discovery [2,3]. The ongoing development of new alternative treatments against fungal infections demonstrates that there is not enough efficacy in the established methods of treatment [4]. Furthermore, current antifungal treatments like the azoles and amphotericin B (AmpB) can have adverse side effects, such as hepatoxicity, due to their pharmacokinetics [5]-[6]. A natural host defense mechanism to counteract infections resides in the antimicrobial peptides (AMPs) produced by nearly every organism. Though some of these possess a nonspecific targeting mechanism and wide antimicrobial spectrum (fungi, bacteria and viruses), their use as systemic agents has been hampered by their low proteolytic stability *in vivo* [7].

Due to the described limitations of AMPs, a newer class of agents based on these naturally occurring agents, called the β-peptides, have gained attention as a method to treat fungal infections [8], [9]. Based on the selection of the β-amino acid (β-AA) subunits, antifungal cationic β-peptides possess a predictable folding behavior as well as an inherent resistance to mammalian proteases [9,10]. Moreover, the rational selection of the β-AA subunits bestows these sequence-directed macromolecules with highly tunable properties; and thus, the β-peptides are considered platform compounds for a new generation of antifungal agents. In order to explore this potential, a number of particular β-peptide models have been successfully tested in fungal pathogens such as *Candida albicans*. However, their toxicity towards mammalian cells remains greatly understudied, a circumstance that has hindered the development of these agents as feasible systemic antifungals [11–13]. Most of the efforts to characterize their effect on host cells have relied on the assessment of hemolysis as a method to evaluate the selectivity of the β-peptides, although the effect of their structural parameters is unknown in other types of mammalian cells. Therefore, their selectivity towards fungi over that of putative cells remains understudied [11,14].

The tunable structural properties of several β-peptides, such as hydrophobicity and helicity, have been related to the β-peptide’s antifungal and hemolytic activities by Palecek, et al [11,15]. They established that the hemolytic activity of the assayed β-peptides increased with hydrophobicity, but the effect of the hydrophobicity on other cell lines remains undefined. Hence, it becomes of utmost importance to assess whether the cytotoxic doses for various mammalian cell lines lie within the therapeutic window needed for efficient fungal inhibition. To provide this much-needed assessment, this work focused on the *in vitro* cytotoxic evaluation of a group of four rationally designed β-peptides with proven antifungal activities on the HepG2 and Caco-2 cell lines. Both cell lines are widely accepted, extensively used to assess the *in vitro* cytotoxicity of xenobiotics, and are crucial for initial comparison studies [16–18]. The immortalized human hepatoma cell line HepG2 is commonly used as a preliminary model of hepatotoxicity as the liver is the main organ responsible for the metabolic processing of xenobiotics. Caco-2 was chosen for this study because this human colon carcinoma cell line is suitable to evaluate the effect of xenobiotic absorption by oral administration routes [19–22].

**Fig 1:**
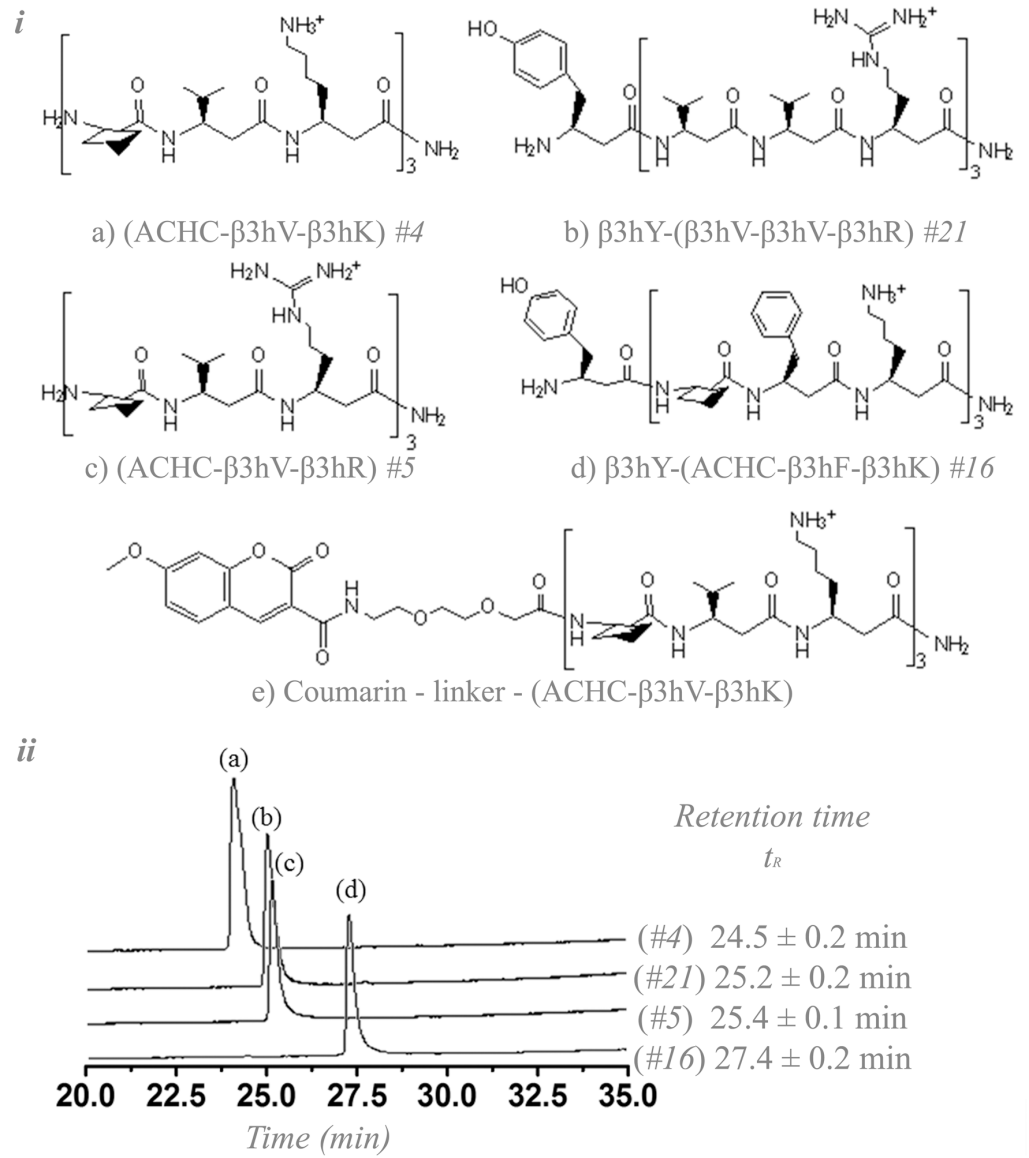
*i*. Structures of antifungal β-peptides used in this study. The codification (#number) corresponds to the nomenclature used by Lee *et al*. in a previous publication [11]. β-peptide (*e*) is a fluorescently labeled β-peptide. The C-terminus is amidated for all β-peptides. *ii*. HPLC-RP retention time for the β-peptides synthesized in this work, see Figs A-D in S1 File for further details.

The four representative β-peptide sequences employed in this study (Fig 1*i*) were selected from a previous baseline study, and the outcomes presented here represent the first effort into exploring the action of these particular sequences on mammalian cell lines. The β-peptides were selected from a battery of 25 β-peptides based on the correlation between their differential hydrophobicity and biological activity against *C*. *albicans* [11]. Notice that the β-peptide *#21* in Fig 1*i*, (consistent with the nomenclature (#number) used by Lee *et al*.) does not have an ACHC residue in its structure, thereby reducing its helicity and influencing its biological activity [23]. Although accessory to the main focus of this paper, analysis of peptide *#21* provides insight for the advancement of this platform and connectivity with previous work. We hypothesized that the selectivity of the β-peptides differs based on the mammalian cell type tested and that it depends on the hydrophobicity of the β-peptide sequences. Dose-response curve analyses for the β-peptides were performed for the epithelial cell models used. Selectivity indices (SI) were computed from these analyses for each sequence. These SIs represent the ratio between the 50% inhibitory concentration (IC50) for the mammalian cells tested divided by the average minimum inhibitory concentration (MIC_aver_) against *C*. *albicans* [24]. Furthermore, we utilized a model antifungal β-peptide that has been widely characterized in *C*. *albicans* to qualitatively and quantitatively study its interaction with Caco-2 and HepG2 through the use of confocal microscopy and cell permeabilization assessments.

## 2. Materials and Methods

### 2.1. Reagents

Amphotericin B (AmpB), ketoconazole (Keto), Phosphate buffer saline (PBS) and trypsin-EDTA solution (0.25%) were purchased from (Sigma-Aldrich, WI, USA).

### 2.2. β-peptides synthesis

A microwave-assisted reaction method was used to synthesize the β-peptides through a solid phase peptide synthesis method (SPPS) using Fmoc protected TentaGel S RAM (Advanced ChemTech, KY, USA) as a matrix resin. According to the β-peptide’s sequence, the following Fmoc protected β-aminoacids were coupled: Fmoc-(*S*,*S*)-trans-2-aminocyclohexanecarboxilic acid (ACHC), Fmoc-L-β-homovaline (β3hV) and Fmoc-L-β-homolysine (Boc) (β3hK) (Peptech, MA, USA). Fmoc-Nω- (2,2,5,7,8-pentamethyl-chromane-6-sulfonyl)-L-β-homo arginine (β3hR), Fmoc-O-tert-butyl-L-β-homotyrosine (β3hY), and Fmoc-L-β-homophenylalanine (hβ3hF) (Chem-Impex International, IL, USA). The quantity of resin (Tentagel) was determined by the scale of the reaction (20–40 μmol).

#### 2.2.1. Synthesis of Coumarin-Linker-(ACHC-β3hV-β3hK)

The methodology used was the same as for microwave assisted SPPS as in Karlsson et al, with some additional steps [14]. After the last β-Lysine of the (ACHC-β3hV-β3hK) was deprotected, the linker {2-[2-(Fmoc-amino)ethoxy] ethoxy}acetic acid (Sigma-Aldrich, WI, USA) was coupled using the same stoichiometry and subsequently deprotected. Then, the reaction vessel was covered with aluminum foil, and 7-methoxycoumarin-3-carboxylic acid (Life technologies, WI, USA) in DMF was added to the coupling reagents master mix. For this step, microwave assistance was not used; instead, the coupling mix was left stirring overnight under laboratory environmental conditions. On the following day, the deprotection process was performed using microwave assistance.

### 2.3. HepG2 and Caco-2 cell culture

HepG2 (passage 7–35) and Caco-2 (passage 51–100) were obtained from American Type Culture Collection (ATCC, VA, USA). Cells were cultured in 25 cm^2^ flasks (Costar, NY, USA) using Dulbecco’s modified Eagle’s medium DMEM (Sigma-Aldrich, WI, USA). DMEM was prepared as reported, but without streptomycin [24]. Cells were maintained at 37°C, 95% relative humidity, and 5% CO2. The medium was changed every other day and cell passages were performed weekly.

### 2.4. Determination of cytotoxicity, principal component analysis (PCA) and selectivity index (SI)

HepG2 and Caco-2 were tittered from a culture at 70–80% confluence. HepG2 and Caco-2 cells were seeded at 13,000 and 9,000 cells, respectively, in a clear flat bottom 96-well plate with dark walls (Corning, NY, USA). Then they were allowed to adhere and proliferate in DMEM for 48 hours. Agents were prepared under the following conditions: β-peptides were dissolved and diluted in DMEM to the final working concentration; while AmpB and Keto were first dissolved in DMSO as a vehicle. The DMSO concentration was kept under 0.5% v/v in the experimental sample as well as in the untreated control cell media [16]. Cells were exposed to the agent during 24 hours. Afterwards, the cells were washed two times with Hank’s Balanced Salt Solution (HBSS) [25]. Then, fresh DMEM was added to each plate and incubated until the control reached approximately 90% confluency. Cell viability was determined using CellTiter Blue^™^ (Promega, WI, USA), an assay based on the capacity of cells to reduce resazurin to resorufin, a fluorescent product whose intensity is proportional to the viable cells in the sample. CellTiter Blue^™^ was prepared in HBSS, and the assay was performed following the protocol suggested by the manufacturer. After incubation, the fluorescence was measured using a Spectra Max Gemini EM spectrofluorometer (560_Ex_/590_Em_) (Sunnyvale, CA, USA). The long-term effect of the treatments with β-peptides was assessed using the model peptide (ACHC-β3hV-β3hK) in a clonogenic assay. In these assays, 1.25x10^5^ and 9x10^4^ cells/ml from HepG2 and Caco-2, respectively, were seeded in a 25cm^2^ flask. The media was changed every two days. Once cells reached a confluence between 70–80%, they were treated in the flask with (ACHC-β3hV-β3hK) at 20 μg/mL in DMEM, a sub-lethal concentration, for 24 hours. They were then washed two times with PBS and allowed to recover in fresh medium for six hours, followed by trypsinization and tittering. 750 HepG2 cells and 500 Caco-2 cells were seeded in a six-well plate (Fisher Scientific, GA, USA). The colony counting was done after sufficiently large colonies were observed in both cell lines [26,27].

*In vitro* cytotoxicity assessments for HepG2 and Caco-2 were performed two independent times, each with four replicates (n = 8). A multivariate study of the data was performed through PCA to ascertain the impact of increasing β-peptide hydrophobicity on the viability of the treated cells. PCA was done using XLSTAT 2014 trial version, under a Pearson approach. The input matrix set to the program consisted of eleven rows and four columns of data. The first column contained the concentrations tested and the remainder columns contained the viability ratio obtained after exposure to the β-peptides *#4*, *#5*, and *#16* (see Fig 1*ii*). The data of β-peptide *#21* was not used in this analysis due to structural differences (lack of ACHC residues), which affects the molecule’s helicity. The ratio IC50/MIC_aver_ represents the selectivity of the β-peptides for the fungal cells over that of host cells, termed selectivity index (SI) [24]. For the purpose of the present study the SIs were calculated based on MICs for *C*. *albicans* published previously [11].

### 2.5. Confocal microscopy

From a cell culture of HepG2 and Caco-2 grown in a 25-cm2 flask at 70–80% confluence, 5x10^4^ cells were seeded in a Nunc^™^ Lab-Tek^™^ Chambered Coverglass 2-wells (Fisher Scientific, GA, USA). They were allowed to adhere and proliferate for 48 hours. Cells were then treated with either fluorescently labeled β-peptide at 16 μg/mL, with non-fluorescent β-peptide at 16 μg/mL or with Triton X-100 at 0.05% v/v as a positive permeation control (Sigma-Aldrich, WI, USA). Treatment with Triton X-100 was achieved only for HepG2, as Caco-2 cells were completely lysed at all concentrations tested, making recovery after washing unsuccessful. Propidium iodide (PI), was used as cell permeabilization indicator. Permeabilization of PI was determined after treatment with either Triton X-100, as a positive control, or β-peptide. For β-peptides, epithelial cells were treated for 1 hour and protected from light. While for Triton treatment, HepG2 cells were incubated for 55 minutes on DMEM, followed by treatment with Triton X-100 for five minutes. Samples were then washed with PBS and stained using a mixture of CellMask^™^ Deep Red Cell Membrane (649_Ex_/667_Em_) (Life Technologies, WI, USA) and Propidium Iodide (540_Ex_/608_Em_) (Sigma-Aldrich, WI, USA) for 30 min following the manufacturer’s protocol, followed by washing three times with PBS. Images were obtained with a spinning disk 3i System (Denver, CO, USA) Olympus IX81 confocal microscope (Olympus America; PA, USA), equipped with a Xenon Fl source for visualization. Images were captured using a 60× oil immersion objective (NA:1.42, RI:0.17) and Rolera EM-C2 camera (SN:Q31153) (Quantitative Imaging Corporation, NC, USA). For confocal image analyses, the experiments were repeated at least three times, and at least two images were obtained for each sample analyzed. All pictures were taken utilizing the same methodology. The Slidebook 5.0 software was used for image acquisition. To minimize the non-specific background associated with experimental variables, at the 408 nm signal, the gamma wavelengths were adjusted to 1.75 for all images. This setting was performed for illustrative purposes; a quantitative analysis was not done for these images.

### 2.6. Flow cytometry for passive transport assessments

Acridine orange (AO) internalization was assessed upon emission of red fluorescence (FL-3 channel) and blue light excitation (488 nm Argon laser). Emissions in the green spectrum (FL-1 channel), indicated DNA binding of AO. For each cell line, a set of two samples (2.5x10^5^ cells each) was prepared: a negative control (untreated cells) and the β-peptide treated sample. Each experiment was replicated at least two times. Cells were resuspended in 5 mL of DMEM. The treatment consisted of a three-hour exposure to (ACHC-β3hV-β3hK) at a concentration of 16 μg/mL. Afterwards, each sample was washed once using PBS and exposed for 4 minutes to a 1 μg/mL AO solution. Cells were later centrifuged, washed, and then the excess supernatant was discarded. The sample was resuspended in the remaining supernatant, approximately 0.3 mL, and loaded onto an Accuri C6 Flow Cytometer (Becton Dickinson, MI, USA). Detection and analysis were performed in the green channel (500–550 nm, FL1 channel) and red channel (600–664 nm, FL3 Channel) fluorescence, which was illuminated with blue light excitation (488 nm).

## 3. Results and Discussion

### 3.1. Cytotoxicity of HepG2 and Caco-2 as a function of β-peptide hydrophobicity

The experiments compiled here establish structural-functional correlations of the β-peptides as a function of the cytotoxicity in mammalian cells other than erythrocytes. Fig 1 depicts the structure of each agent assayed and demonstrates the sorting of the peptides according to their retention times (t_R_), as previously published [11,28,29]. This assessment resulted in Fig 1*ii* where *#4* is the least hydrophobic β-peptide, followed by *#21* and *#5*, which exhibit comparable hydrophobicities, and finally *#16* as the most hydrophobic one.

**Fig 2:**
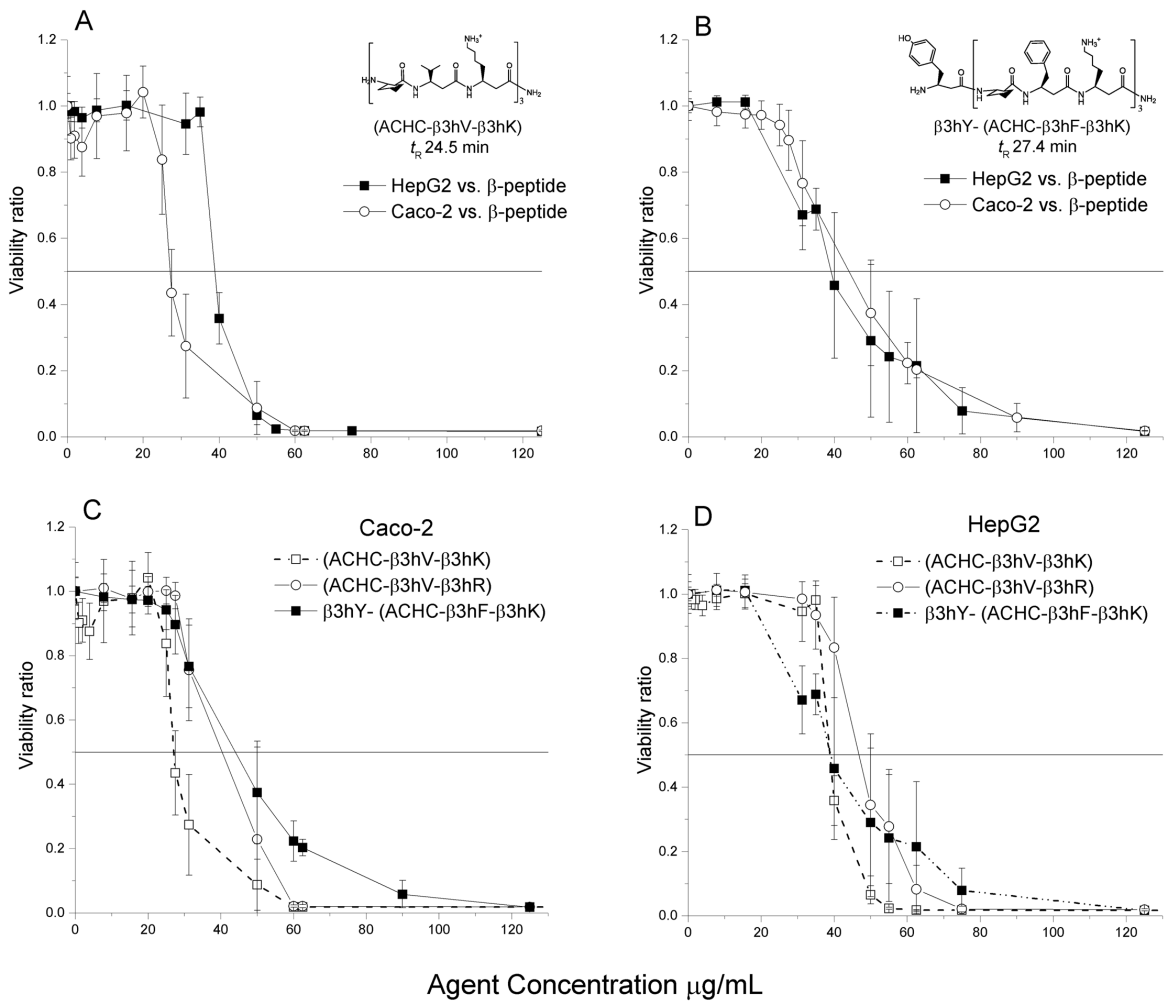
Dose effect curves. Panel A and B represent the curves for HepG2 (▪) and Caco-2 (o), exposed to the least and most hydrophobic β-peptide (*#4* and *#16*, respectively) during 24 hours respectively. Panels C and D feature the behavior of Caco-2 and HepG2 correspondingly, for the three ACHC-containing β-peptides. The curves represent the mean of two independent experiments, each with four biological replicates. The error bars correspond to the standard error of the mean, where n = 8.

The cytotoxic effects caused by the chosen β-peptide sequences are exhibited in the dose-effect curves (viability ratio vs. agent concentration) in Fig 2. Comparison of panels A (β-peptide *#4*, the least hydrophobic β-peptide) and B (β-peptide *#16*, the most hydrophobic β-peptide) show a differential response between both cell lines assayed. The peptides in A and B were previously characterized as globally amphiphilic and they both have a stable helical structure due to the presence of the ACHC residues in the sequence repeat. The difference in t_R_ is due to the substitution of the residue that lies on the β-peptide’s polar side, which was changed from a β-valine (peptide *#4* in A) to a β-phenylalanine (peptide *#16* in B). Additionally, the β-peptide in panel B contains a β-tyrosine in the N-terminal. These changes have been reported to increase the hydrophobicity of the structure. Panel A shows that Caco-2 responds with more susceptibility to the least hydrophobic peptide than HepG2. Interestingly, a difference between the behaviors of both cell lines was not observed for β-peptide #16, the most hydrophobic peptide of the group. The effect of every differentially hydrophobic peptide is shown in panels C and D, and a significant shift to the right of the curves is clearly appreciated for Caco-2 as hydrophobicity increases. A shift to the right indicates a reduction in the cytotoxicity of the cell line. Conversely, this displacement was subtle for HepG2 (D).

Furthermore, the cytotoxicity of two currently used antifungal agents, Keto and AmpB, was assessed as a point of reference (Fig E in S1 File). For HepG2, Keto was relatively less toxic (IC50 = 70 μg/mL) when compared to the β-peptides (IC50s 39–52 μg/mL). Interestingly, AmpB did not reach 100% cytotoxicity of HepG2 at the tested concentrations even though it showed signals of toxicity at lower concentrations; this behavior at low concentrations is expected given its reported hepatotoxicity [30]. The dose-effect curves for HepG2 also demonstrated that for a concentration of 30 μg/mL three of the four β-peptides did not exhibit significant cytotoxicity, while AmpB caused 30% inhibition of the cells at this concentration (IC30 = 30 μg/mL). The most hydrophobic β-peptide, β3hY-(ACHC-β3hF-β3hK), presented the greatest toxic effect at these lower concentrations, even more toxic than AmpB in HepG2. The difference among dose-effect curves for the tested β-peptides might indicate potential mechanistic differences in their ability to affect cell viability. Although the β-peptides are still more cytotoxic than the reference agents overall, locally, there are points where their dose response curves converge. Particularly at low concentrations, the overlap of the curves is notable. Moreover, our group has found that β-peptides can be used at low concentrations for the potentiation of current agents, thus validating the relevance of the β-peptide platform and boosting further research interests [13].

The dose effect curves for HepG2 depicted in Fig E in S1 File, were used for comparison with the level of toxicity exhibited by Caco-2 upon exposure to selected β-peptides in the same chart. The behaviors obtained for Caco-2 exposed to Keto was not statistically different than the response observed for HepG2. AmpB did not affect the viability of Caco-2 at the concentration range tested. This result corresponds to the inability of the hydrophobic AmpB molecule to interact with the polar membrane environment of the Caco-2 model [31]. The curves pertaining to Keto or AmpB against Caco-2 were omitted from Fig E in S1 File, for simplicity. Comparable observations were previously reported about hydrophobic α-peptides and their limited transport across the intestinal lining [32,33]. Fig 2 illustrates that β-peptides were more harmful to Caco-2 (IC50 = 27–45 μg/mL) when compared to HepG2 (IC50 = 38–52 μg/mL). We noticed an inverse correlation between the hydrophobicity of the β-peptide and the cytotoxicity for Caco-2 since the most hydrophobic β-peptide was less harmful to this cell line; a behavior similarly described previously for AmpB. The most hydrophobic β-peptide, β3hY-(ACHC-β3hF-β3hK), caused the lowest cytotoxicity (IC50, = 45 μg/mL), and as the β-peptide concentration increased the viability of both models leveled off, ending in higher IC90 than the rest of the β-peptides. Whereas the least hydrophobic β-peptide, (ACHC-β3hV-β3hK), caused the highest cytotoxicity (IC50 = 27 μg/mL). It is important to mention that for concentrations below 20 μg/mL, the tested β-peptides did not exert any significant effect in the viability of any of the cell lines; the reported minimum inhibitory concentrations (MIC) for these β-peptides against *C*. *albicans* are all below 20 μg/mL.

**Fig 3:**
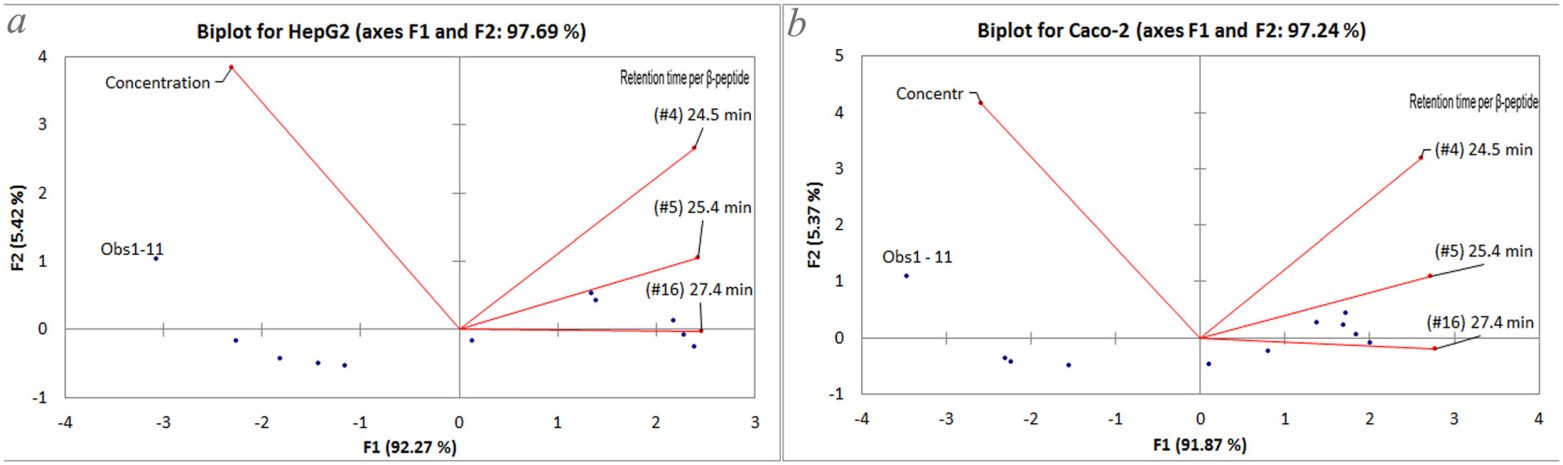
PCA of β-peptide concentration and viability of cells by each different hydrophobic β-peptides. Panels *a* and *b* are for HepG2 and Caco-2, respectively.

To conclusively establish the effect of the hydrophobicity on cell viability, a PCA chart was constructed, see Fig 3.

From the matrix input into the XLSTAT, four main components were found. From these, the information was mainly being modeled by the component F1; up to 92% and 91% for HepG2 and Caco-2, respectively. Moreover, the sum of the components F1 and F2 represented around 97% of the data. The plot evidences that the most hydrophobic β-peptide had a greater impact on F1. Both charts demonstrate that increasing retention time increases the effect on the principal component. This leads to the conclusion that there is an association between the β-peptide’s hydrophobicity and viability.

The β-peptide β3hY-(β3hV-β3hV-β3hR) demonstrated the lowest cytotoxicity (highest IC50) in HepG2 even though it was not the least hydrophobic β-peptide, see Fig E in S1 File. This is presumably due to the absence of the ACHC side chain in its structure, which decreases the helicity percentage of the peptide. The helicity parameter of each β-peptide sequence had previously been associated with biological activity towards *C*. *albicans* [11]. Interestingly, the difference in the IC50 between Caco-2 and HepG2 for the peptide β3hY-(β3hV-β3hV-β3hR) was the greatest among the β-peptides tested. That is, β3hY-(β3hV-β3hV-β3hR) was the least cytotoxic peptide for HepG2, but behaved in an opposite manner in Caco-2.

Subsequently, the relationship between mammalian cytotoxicity and yeast inhibition in the context of our group of β-peptides was explored using the selective index (SI) [24].

Table 1 encompasses the described observations and the SIs computed in this work. The SIs obtained for β-peptides (*a*) and (*b*) are effectively equal for both cell models. However, the SI increased considerably with hydrophobicity, as seen for the other two β-peptides (*c* and *d*). It is important to point out that the SI for erythrocytes, calculated from the data in Palecek *et al*, decreased with hydrophobicity [11]. This proved to be opposite to the trend observed in Caco-2 and HepG2. These previously unseen results must be highlighted because they indicate that the generally desired scenario, the broadening of the gap between cytotoxic effects and antifungal effectiveness against *C*. *albicans*, was achieved by increasing the β-peptide’s hydrophobicity.

**Table 1:** β-peptides selected for the study and analysis of their cytotoxicity towards HepG2 and Caco-2 cells compared to their antifungal activities.

	β-peptide sequences (from lower to higher hydrophobicity level)	IC50 (μg/mL)	IC50 (μg/mL)	SI	SI
		HepG2	Caco-2	HepG2	Caco-2
(I)	ACHC-β3hV-β3hK **(*#4*)**	38.75 ± 0.89	27.33 ± 1.5	3.23 ± 0.07	2.28 ± 0.13
	ACHC-β3hV-β3h-R **(*#5*)**	47.25 ± 5.12	43.33 ± 9.0	5.91 ± 0.64	5.41 ± 1.13
	β3hY-(ACHC-β3hF-β3hK) **(*#16*)**	42.5 ± 6.21	53.17 ± 5.74	7.01 ± 1.04	7.53 ± 0.96
(II)	β3hY-(β3hV-β3hV-β3hR) **(*#21*)**	52.5 ± 4.98	31.33 ± 3.27	3.28 ± 0.31	1.96 ± 0.20

The IC50 for HepG2 and Caco-2 were determined after 24 hours of exposure. The Selective Index (SI = IC50/MIC_aver_) represents the quantitative measurement of the gap between the IC50 and the average MIC, meaning that higher SI values are indicative of selectivity towards *C*. *albicans* over the mammalian cell lines tested [11]. Average MICs used for SI calculations were previously reported by Palecek et al. [11], [14]. The uncertainty in the SI values were calculated using propagation of error formulas.

As mentioned previously, β-peptide #21 does not contain an ACHC residue. This affects the helical conformation, but offers potential benefits regarding its synthesis since the addition of an ACHC residue to a peptide sequence decreases the synthesis efficiency [34]. Although peptides #21 and #5 have similar t_R_ values, the SI values varied suggesting a negative effect on the selectivity of the peptide. Upon inspection of this parameter, it is notable that the IC50s for both cell lines are similar for both peptides but the MIC for peptide #21 increased to twice that of #5 causing a decrease in the SI. This observation implies that helicity affects mammalian cell lines tested in a different manner than it does yeast.

The long-term cytotoxic effect of β-peptides was evaluated using clonogenic assays (see Fig F in S1 File) [35]. In order to validate the data obtained from the dose-response curves for Caco-2 and HepG2, (ACHC-β3hV-β3hK) was selected as a β-peptide model to perform these assays due to its well-established biological activity against *C*. *albicans*, its hemolytic activity, and, with this study, its effect on HepG2 and Caco-2. A constant concentration of 20 μg/mL was chosen for these experiments. The chosen concentration of (ACHC-β3hV-β3hK) falls within the range of concentrations used in short-term assays, where 20 μg/mL of this β-peptide assured that both of the cell lines were affected to the same extent. Fig F in S1 File. indicates that there is no significant difference between the surviving fraction estimated for long-term effect and the viability obtained previously with a short-term cytotoxicity assay. The slight decrease in colony forming ability of Caco-2 in the clonogenic assay at 20 μg/mL when compared to untreated controls falls within the error observed in the corresponding dose-effect curves. These results rule out other effects that could not be observed in cytotoxic analysis, such as DNA damages which could involve apoptotic response appreciated in a long-term growth defect [26,27,36].

### 3.2. Confocal microscopy shows cellular localization of fluorescently labeled β-peptide

**Fig 4:**
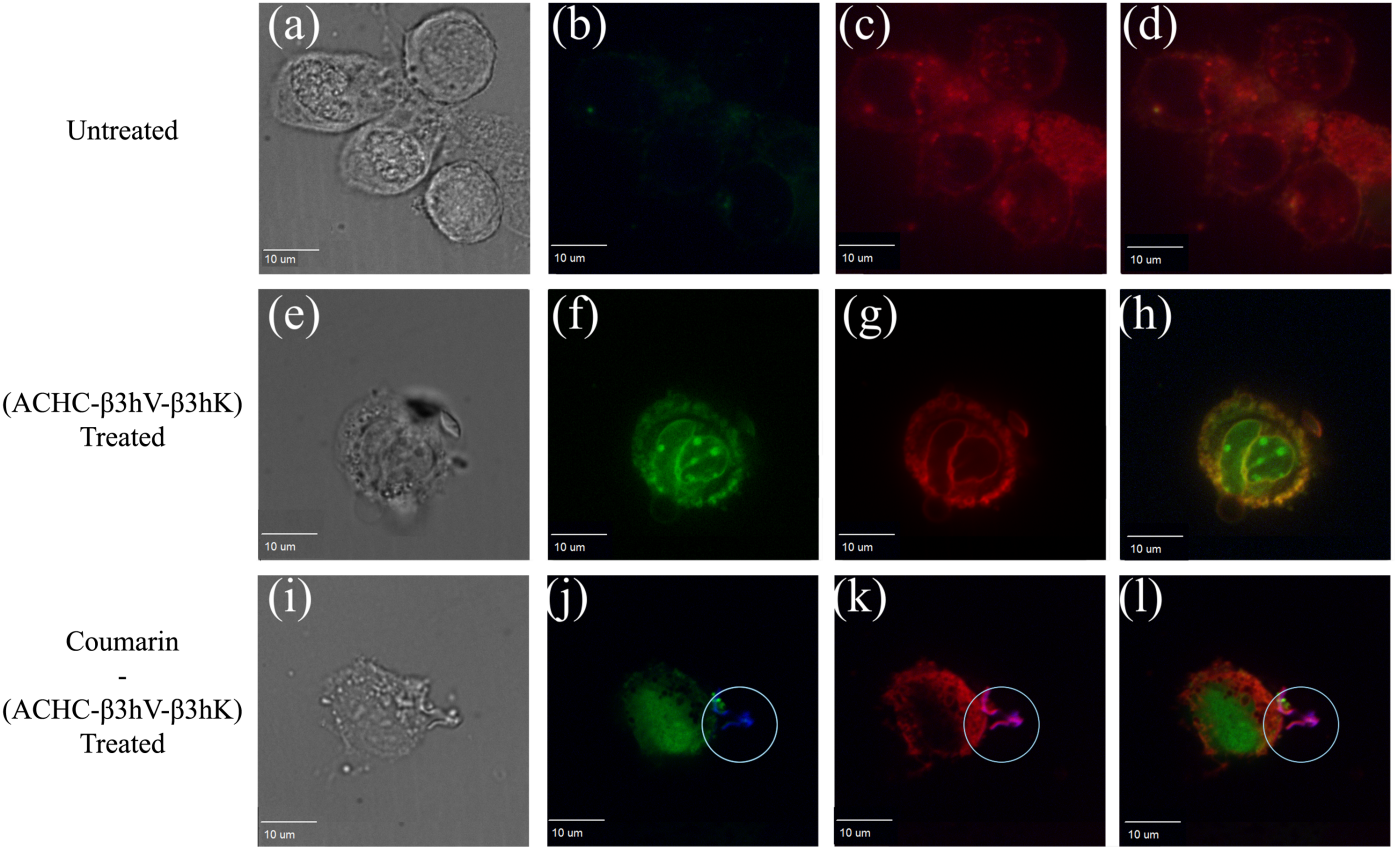
Confocal images for Caco-2 cells. (a-d) untreated cells, (e-h) cells treated with 16 μg/mL of (ACHC-β3hV-β3hK), and (i-l) cells treated with 16 μg/mL of coumarin-(ACHC-β3hV-β3hK). (a, e, i) show phase-contrast images, (b, f, j) show the signal emission for the 545 (green) and 405 (blue) nm filters, (c, g, k) show signals for the 640 (red) and 408 (blue) nm filters, and (d, h, l) show all signals. These are representative images of at least three independent experiments. The exposure time was 1 hour. Cells were stained for 30 min as described in the methodology.

Confocal microscopy was utilized to qualitatively visualize the localization of a fluorescently labeled β-peptide within the cellular environment. Fig 4 illustrates representative images obtained from Caco-2 stained with fluorescent dyes for nucleic acids and the cell membrane, while exposed to a fluorescently labeled β-peptide (panels i-l). The green color, corresponding to the signal obtained with the 545 nm filter, is used for propidium iodide (PI). This fluorescent molecule has binds to nucleic acids, and its detection is indicative of cell membrane permeation. The red color was assigned to the signal obtained with the 640 nm filter and represents the deep red cell membrane dye. The blue signal (408 nm filter) is the coumarin-linked (ACHC-β3hV-β3hK), the fluorescent antifungal β-peptide.

Panels (a-d) in Fig 4 depict phase contrast and fluorescent micrographs of untreated Caco-2 cells with all dyes. They illustrate intact cell membranes, evidenced by the inability of PI to interact with intracellular nucleic acids. Conversely, PI-labeling of the nucleic acids was observed in conditions where β-peptide was present (panels f-h and j-l). Panels (e-h) illustrate the exposure of Caco-2 to the unlabeled (ACHC-β3hV-β3hK). This treatment was performed to establish the effect of the non-fluorescent β-peptide and thus ensure that the presence of a coumarin label and linker did not alter this effect. No additional signals were observed under these conditions, other than the signal for PI. The permeabilization of PI was evident with and without the coumarin-linker label, both to a similar extent, which ruled out that the dyes caused a false positive. Panels (i-l) display that the β-peptide remained associated to the cell membrane. These panels substantiate that the β-peptide and the cell membrane were overlapped as though they are complexed and projected from the cell surface (encircled). Additionally, no evidence of β-peptide interaction with the molecules stained with PI was found. These results show an affinity for the cell membrane with respect to other internal cellular structures, supporting that the cell permeabilization was caused by the peptide.

**Fig 5:**
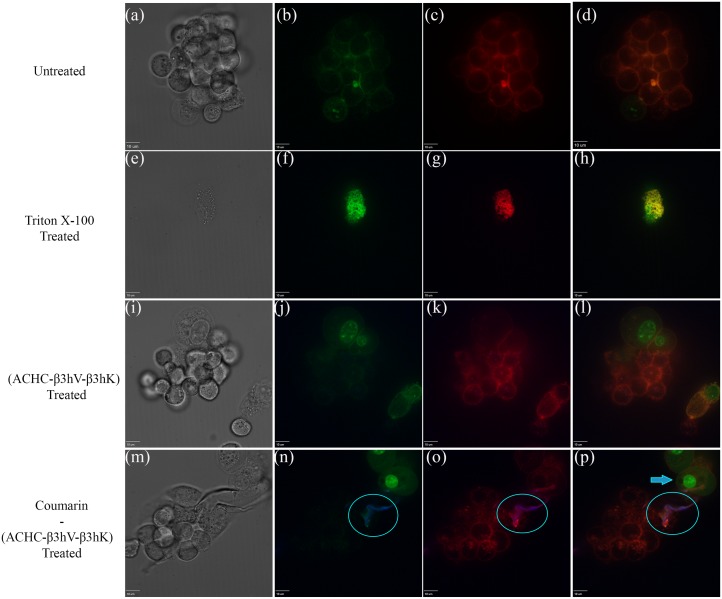
Confocal images for HepG2. (a-d) untreated cells, (e-h) cells treated with Triton X-100 at 0.05% v/v, (i-l) cells treated with 16 μg/mL of (ACHC-β3hV-β3hK), and (m-p) cells treated with 16 μg/mL of coumarin-(ACHC-β3hV-β3hK). (a, e, i, m) show phase-contrast images, (b, f, j, n) show signal emissions for the 545 (green) and 405 (blue) nm filters, (c, g, k, o) show signals for the 640 (red) and 408 (blue) nm filters, and (d, h, l, p) show all signals. The oval encircles a zone where the β-peptide was detected, and co-localized with cell membrane, and the arrow points to the nucleus of the cell affected by the β-peptide.

Fig 5 illustrates the emission signals obtained with HepG2 cells that were treated and stained under the same methodology described for Caco-2. For this assay Triton 100-X was used as a positive control for cell permeabilization, to compare its effect to that of the β-peptide on treated cells. Panels (a-d) represent the images of untreated cells. Phase contrast is illustrated in (a) and stained images appear in (b-d), which show an unstained nucleus since the cell membrane remained intact.

**Fig 6:**
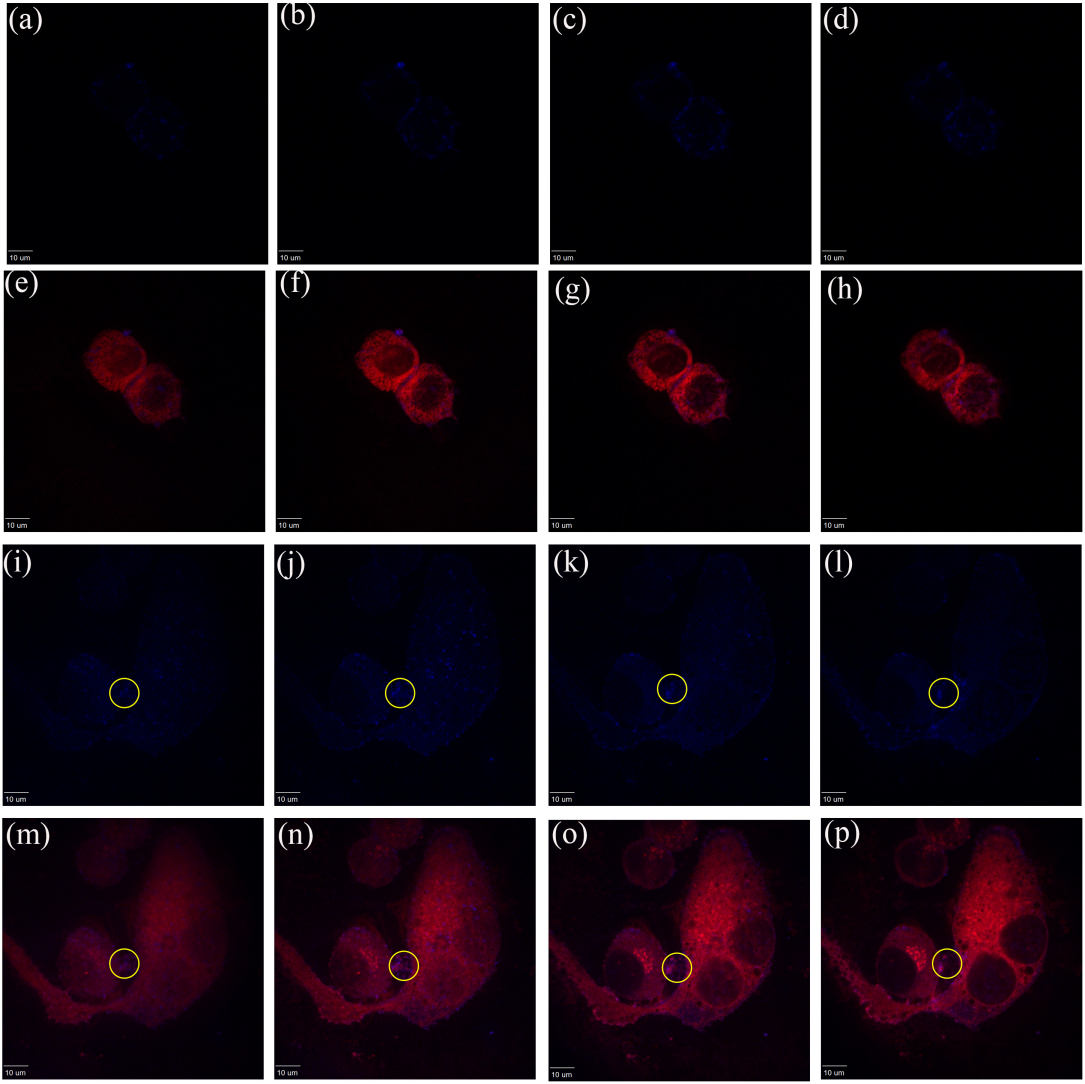
HepG2 exposure for 1 hour to 16 μg/mL of coumarin-(ACHC-β3hV-β3hK). The images were captured increasing Z to 0.5 μm from left to the right per plane. The experiments were conducted at least two independent times. The blue represents coumarin signal at 408 nm captured. Panels (e-h & m-p) show coumarin (blue) signals co-localization with structures stained with the cell membrane dye (red).

The phase contrast images depict how HepG2 cells have a tendency to form small colonies, as opposed to Caco-2. Panels (e-h) represent the treatment with Triton 100-X. These images display permeated cells whose nucleic acids were stained with undefined nuclei, probably because the Triton was concentrated enough to affect the nuclear integrity. This control was attempted in Caco-2 but the Triton completely detached the cells making it impossible to recover treated cells. Panels (i-l) represent the effect on cells treated with non-fluorescent (ACHC-β3hV-β3hK) as an additional positive control. This β-peptide shows the effect on the cell membrane by evaluating PI staining of the nucleic acids. The trial resulted in disrupted cells with well-defined stained nuclei; this is also shown in panels (m-p). In panels (m-p) it can also be observed that, as in Caco-2, the coumarin-linked (ACHC-β3hV-β3hK) attaches to the cell membrane. In image (p), encircled by the oval, the coumarin-labeled peptide was co-localized with a disrupted cell membrane, evidenced by the green nuclear stain (arrow). Meanwhile, Fig 6 represents images from the cell membrane surface and with three additional images advancing 0.5 μm in the visual plane. Both (a) and (b) show HepG2 treated with a concentration of 16 μg/mL of coumarin-labeled (ACHC-β3hV-β3hK), an amount of β-peptide that does not exert a significant effect on viability, but that does cause cell membrane permeation. Images on (a-d) and (i-l) panels, illustrate spots of coumarin signal primarily located on the cell membrane surface. Evidence of potential peptide internalization is observable through a number of blue spots in the cytoplasmic area, within the cell body. Panels (e-h) and (m-p) display that the coumarin signal co-localized with the cell membrane dye. Blue dots were observed on the membrane surface, and projections from the cell surface showed that the peptide remained associated with it (yellow ovals). These results suggest that the coumarin signal overlapped with the membrane dye and that it also crossed to sections within the cell.

Confocal microscopy evidenced growth and morphological differences between the cellular models (untreated controls in Figs 4 and 5). These differences might explain the differential cellular responses to the β-peptides observed in dose-effect curves. Cellular polarity determined by the organization and arrangement of cell membrane proteins is one such difference. Caco-2 cells possess a typical simple polarity characterized by a columnar morphology, where top and bottom surfaces communicate and lateral walls participate in cell-cell interactions. This is common to monolayered epithelial cells. HepG2, however, possesses a very different morphology of complex polarity with a polygonal shape characterized by the formation of multiple grooves between nearby cells [31]. These morphological differences might impact the extent of the dose response related to cell density. The cell density dependent toxicity of both cell models was further characterized using clonogenic assays and is reported in Fig G in S1 File. We found that single HepG2 cells are more susceptible than Caco-2 upon exposure to β-peptides.

### 3.3. Indirect cell membrane passive transport assessments upon exposure with β-peptide (ACHC-β3hV-β3hK)

**Fig 7:**
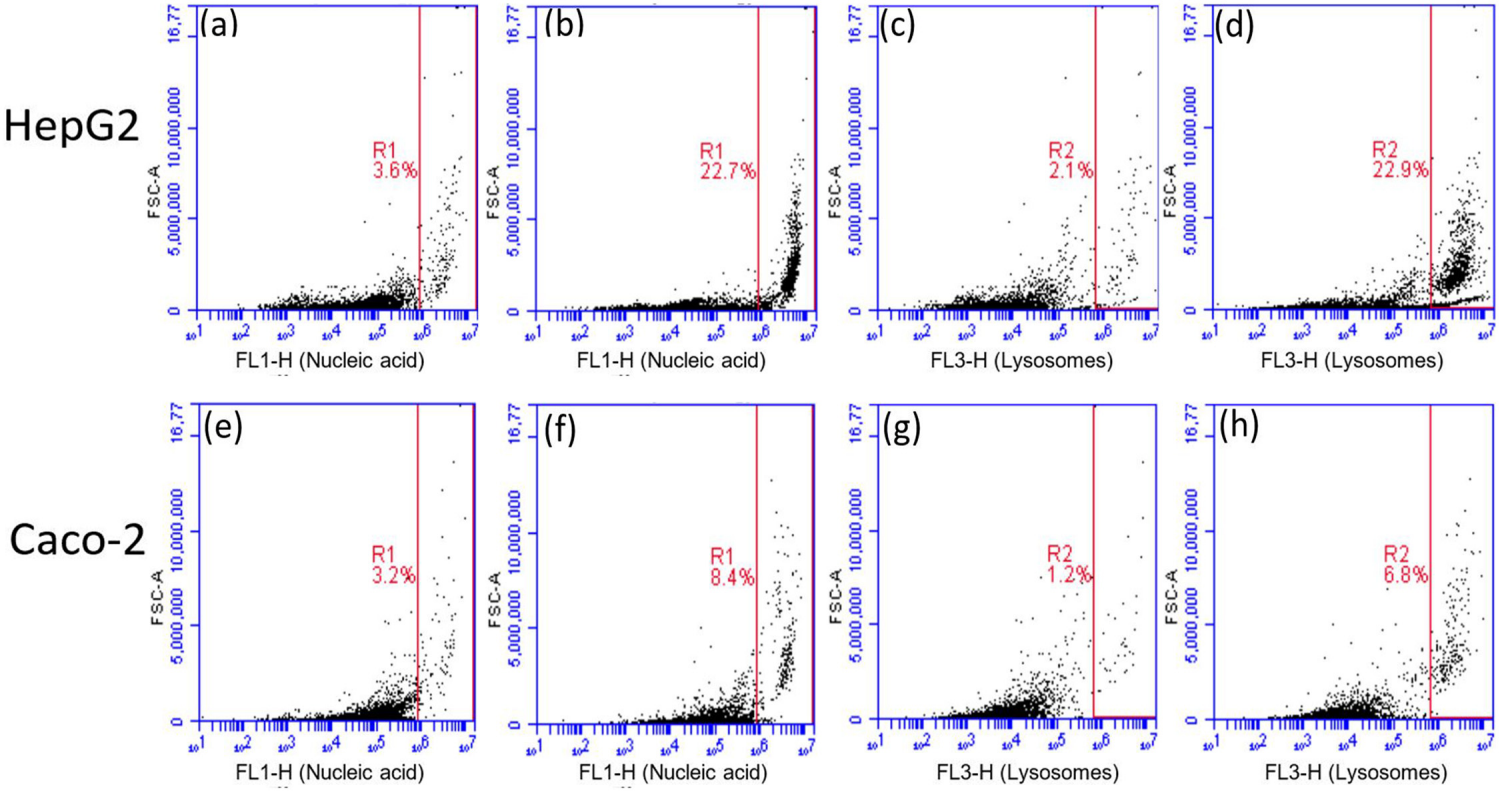
Acridine orange (AO) uptake in HepG2 and Caco-2. The (a, c, e & g represents untreated cells, and (b, d, f and h) represents cells treated with 16 μg/mL of (ACHC-β3hV-β3hK) for 3 hours in suspension under incubation conditions. FL1-H represents nucleic acids stained with AO while FL3-H represents lysosomal uptake of AO. The experiment was performed at least two independent times and the measurement has two biological replicate the figure presented is a representative result obtained from the assays performed.

Microscopy results are consistent with a cell permeabilization action for the β-peptide tested. To gain further insights into this action, transport across the cell membrane in mammalian models upon β-peptide exposure was assessed using flow cytometry as described by Alvarez-Berríos, et al [25] (Fig 7). The experiment consisted in quantifying the overall percentage of cell population, treated with β-peptide, which internalized the fluorescent dye acridine orange (AO) and compared to the extent of internalization in the population of untreated cells. AO is a lysosomotropic dye permeable to the cell, and a metachromatic fluorochrome that emits a red fluorescence (FL-3 channel) when excited by a blue light (488 nm argon laser) [25,37,38]. However, it also binds to DNA in which case it emits in the green spectrum (FL-1 channel). The uptake of AO was monitored for the same number of events with HepG2 and Caco-2. Signals for both lysosome- and DNA-bound AO were obtained. Panels (a-b) are for DNA-bound AO signals (FL1H) and panels (c-d) are for lysosome-bound AO signals (FL3H) in HepG2. In the same figure, Caco-2 panels (e-f) and panels (g-h) use the same configuration as (a-b) and (c-f), respectively. Panels (a), (c), (e), and (g) are untreated samples (controls) and all remaining panels represent samples treated with 16 μg/mL of (ACHC-β3hV-β3hK). The regions labeled as R1 or R2 delimit the population percentages that display the AO signal from the rest of the events. Both experiments were performed under identical conditions for untreated and β-peptide-treated cells. The results indicated that the uptake of AO increases when the mammalian cells were treated with the β-peptide for all experiments. Results demonstrated that mammalian cells treated with (ACHC-β3hV-β3hK) exhibited enough altered membrane properties to enhance the uptake of AO in both cell models used. In HepG2, a higher percentage of the population had increased AO uptake after treatment than that encountered with Caco-2. (Fig 7). However, for HepG2 this particular peptide exhibited less cytotoxicity. This observation may indicate that mechanisms of cytotoxicity may involve additional targets other than the cell membrane.

## 4. Conclusions

The present studies offer evidence that the hydrophobicity of the β-peptides is connected to their selectivity towards HepG2 and Caco-2. This physicochemical parameter is also responsible for the β-peptide’s fungicidal effect towards *C*. *albicans*. Hydrophobicity could therefore be a basis for the selection of a candidate β-peptide since both selectivity and biological activity are necessary for therapeutical purposes. Previous studies have shown that increasing hydrophobicity resulted in a more hemolytic β-peptide; however we found that selectivity indices increased with hydrophobicity in both Caco-2 and HepG2. The results seen on HepG2 and Caco-2 prove our hypothesis that hemolysis studies should be complemented with cytotoxicity assays on putative mammalian cells, and that *in vitro* hemolysis data may not necessarily be representative of all host cells. These differences could be explained given the differences between these cells in terms of cellular architecture, membrane composition, hydrophobicity or charge, which is directly related to their particular function. Since the SIs for all peptides were greater than one, a window of improvement for these antifungal materials as therapeutic developmental templates widens.

## Supporting Information

S1 File **Fig A in S1 File. MALDI-TOF-MS Calculated for NH**_**2**_**-(ACHC-*β***^**3**^**hVal-*β***^**3**^**hLys)**_**3**_, **(#4)**. Expected mass for C_60_H_111_N_13_O_9_, [M+H]^+^: 1158.9. Peak found at 1158.9. **Fig B in S1 File. MALDI-TOF-MS Calculated for NH**_**2**_**-(ACHC-*β***^**3**^**hVal-*β***^**3**^**hArg)**_**3**_, **(#5)**. Expected mass for C_60_H_111_N_19_O_9_, [M+H]^+^: 1242.9. Peak found at 1242.9. **Fig C in S1 File. MALDI-TOF-MS Calculated for NH**_**2**_-***β***^**3**^**hTyr-(*β***^**3**^**hVal-*β***^**3**^**hVal-*β***^**3**^**hArg)**_**3**_, **(#21)**. Expected mass for C_67_H_122_N_20_O_11_ [M+H]^+^:1384.0. Peak found at 1384.4. **Fig D in S1 File. MALDI-TOF-MS calculated for NH**_**2**_-***β***^**3**^**hTyr-(ACHC-*β***^**3**^**hPhe-*β***^**3**^**hLys)**_**3**_, **(#16)**. Expected mass for C_82_H_122_N_14_O_11_ [M+H]^+^: 1480.0. Peak found at 1479.5. **Fig E in S1 File. Dose effect curves for HepG2 (▪) and Caco-2 (O), exposed to a battery of β-peptides during 24 hours, correlated to dose effect curves for (▫) Amphotericin B and (x) Ketoconazole**. The graphs were arranged based on the hydrophobicity of the β-peptide, which increases alphabetically. This is the mean of two independent experiments, each with four biological replicates. The error bars correspond to the standard error of the mean n = 8. **Fig F in S1 File. Clonogenic capacities of HepG2 and Caco-2 after (ACHC-β3hV-β3hK) exposure**. Treatment underwent for 24 hours at 20μg/mL β-peptide. The assay was performed two independent times, each with three biological replicates. The plating efficiency was > 9.5% for HepG2 and > 22% for Caco-2 for treated cells. *Statistic analysis used was described by Gupta et al [5].** 2-Sample t-Test was used to establish that the means are statistically different. **Fig G in S1 File. Cell density-dependent toxicity**. 750 and 500 cell were trypsinized and seeded per well respectively. After 48 hours, both were exposed to 16 μg/mL of (ACHC-b3hV-b3hK) for 24 hours. Cell viability was determined by CFU counting. The lowest plating efficiency obtained for HepG2 was 4% and the lowest for Caco2 was 10%. The statistical analysis was performed as described by Gupta et al [5].
(PDF)
