# Correction: Hydrophobicity of Antifungal β-Peptides Is Associated with Their Cytotoxic Effect on *In Vitro* Human Colon Caco-2 and Liver HepG2 Cells

**DOI:** 10.1371/journal.pone.0157025

**Published:** 2016-06-02

**Authors:** Camilo Mora-Navarro, Janet Méndez-Vega, Jean Caraballo-León, Myung-ryul Lee, Sean Palecek, Madeline Torres-Lugo, Patricia Ortiz-Bermúdez

The following information is missing from the Funding section: This work was partially supported by NIH grants U54 CA 96300/u54CA96297 and 1R01 AI092225 to MTL and SPP respectively. This work was also partially supported by Agriculture and Food Research Initiative Competitive Grant No. 2012–01871 from the USDA National Institute of Food and Agriculture, Hispanic Serving Institution to POB.

The following information is missing from the Acknowledgements section: Thank you to Michael Álvarez-Navarro from the University of Puerto Rico at Mayagüez for assistance in statistical analyses.
