# Supplementary material for: Hydrophobicity of Antifungal β-Peptides Is Associated with Their Cytotoxic Effect on In Vitro Human Colon Caco-2 and Liver HepG2 Cells
Source: PLoS One. 2016 Mar 18;11(3):e0149271. doi: 10.1371/journal.pone.0149271 (PMC4798767; doi:10.1371/journal.pone.0149271)
Supplement: S1 File — Fig A in S1 File. MALDI-TOF-MS Calculated for NH2-(ACHC-β3hVal-β3hLys)3, (#4). Expected mass for C60H111N13O9, [M+H]+: 1158.9. Peak found at 1158.9. Fig B in S1 File. MALDI-TOF-MS Calculated for NH2-(ACHC-β3hVal-β3hArg)3, (#5). Expected mass for C60H111N19O9, [M+H]+: 1242.9. Peak found at 1242.9. Fig C in S1 File. MALDI-TOF-MS Calculated for NH2-β3hTyr-(β3hVal-β3hVal-β3hArg)3, (#21). Expected mass for C67H122N20O11 [M+H]+:1384.0. Peak found at 1384.4. Fig D in S1 File. MALDI-TOF-MS calculated for NH2-β3hTyr-(ACHC-β3hPhe-β3hLys)3, (#16). Expected mass for C82H122N14O11 [M+H]+: 1480.0. Peak found at 1479.5. Fig E in S1 File. Dose effect curves for HepG2 (▪) and Caco-2 (O), exposed to a battery of β-peptides during 24 hours, correlated to dose effect curves for (▫) Amphotericin B and (x) Ketoconazole. The graphs were arranged based on the hydrophobicity of the β-peptide, which increases alphabetically. This is the mean of two independent experiments, each with four biological replicates. The error bars correspond to the standard error of the mean n = 8. Fig F in S1 File. Clonogenic capacities of HepG2 and Caco-2 after (ACHC-β3hV-β3hK) exposure. Treatment underwent for 24 hours at 20μg/mL β-peptide. The assay was performed two independent times, each with three biological replicates. The plating efficiency was > 9.5% for HepG2 and > 22% for Caco-2 for treated cells. *Statistic analysis used was described by Gupta et al [5].** 2-Sample t-Test was used to establish that the means are statistically different. Fig G in S1 File. Cell density-dependent toxicity. 750 and 500 cell were trypsinized and seeded per well respectively. After 48 hours, both were exposed to 16 μg/mL of (ACHC-b3hV-b3hK) for 24 hours. Cell viability was determined by CFU counting. The lowest plating efficiency obtained for HepG2 was 4% and the lowest for Caco2 was 10%. The statistical analysis was performed as described by Gupta et al [5]. (PDF) [file pone.0149271.s001.pdf]

S 1. *β*-Peptide synthesis. *β*-Peptides were synthesized using the reported TentaGel (20-40 μmol) microwave-assisted solid phase peptide synthesis [1]–[4]. The coupling reagents included *O*-benzo-triazol-1-y1-*N,N,N'*,N'-tetramethyluroniumhexafluoro phosphate (HBTU), 1-Hydroxybenzotriazole (HOBt), and the secondary amine *N,N*-diidopropyl ethylamine (DIEA) (Advanced Chemtech, KY, USA) which were dissolved in *N,N* dimethyl formamide (DMF) at 0.5 M, 0.5 M, and 1 M, respectively, for each coupling reagent. The coupling step began with a master mix of coupling reagents and the DIEA. The first β-aminoacid, previously dissolved in DMF, was added to the coupling master mix and then moved to an Extract-Clean Column (GRACE, IL, USA). The MARS5 CEM microwave reactor was used to accelerate the coupling/deprotection steps. The deprotection was performed by adding a 20% v/v mix of piperidine in DMF. Washings took place between each coupling and deprotection step in a sequence of three times for each one of the following solvents: DMF, dichloromethane (DCM) and DMF. Once the deprotection for the final β-aminoacid of the sequence was done, the washing step was repeated five times for each solvent again. The deprotected β-peptide bound to the resin was washed in DCM and cleaved from the resin using a solution of triisopropyl-silane (2.5% v/v), water (2.5% v/v) and trifluoroacetic acid (TFA) (95% v/v) for two hours with stirring. The β-peptide was purified by preparative RP-HPLC fitted with a C-18 column, using a gradient ranging from 25-73% (v/v) for 40 minutes beginning at 10% acetonitrile, mobile phase A, and 90% water, mobile phase B, each containing 0.1% TFA. The product was confirmed by MALDI-TOF mass spectrometry. see S1- 4 Fig.

S 1.1 Hydrophobicity characterization. The β-peptides were dissolved in a 80:20 water:acetonitrile solution at 0.5 μg/mL. The column used was a C18 reverse phase, waters X-bridge, BEH130, 5um, 4.6x250mm at 1 mL/min flow rate. Using a gradient program from 20-70 % of acetonitrile in water, both with TFA at 1% v/v. The RP-HPLC chromatogram is

presented in Fig. 1 panel *ii*.

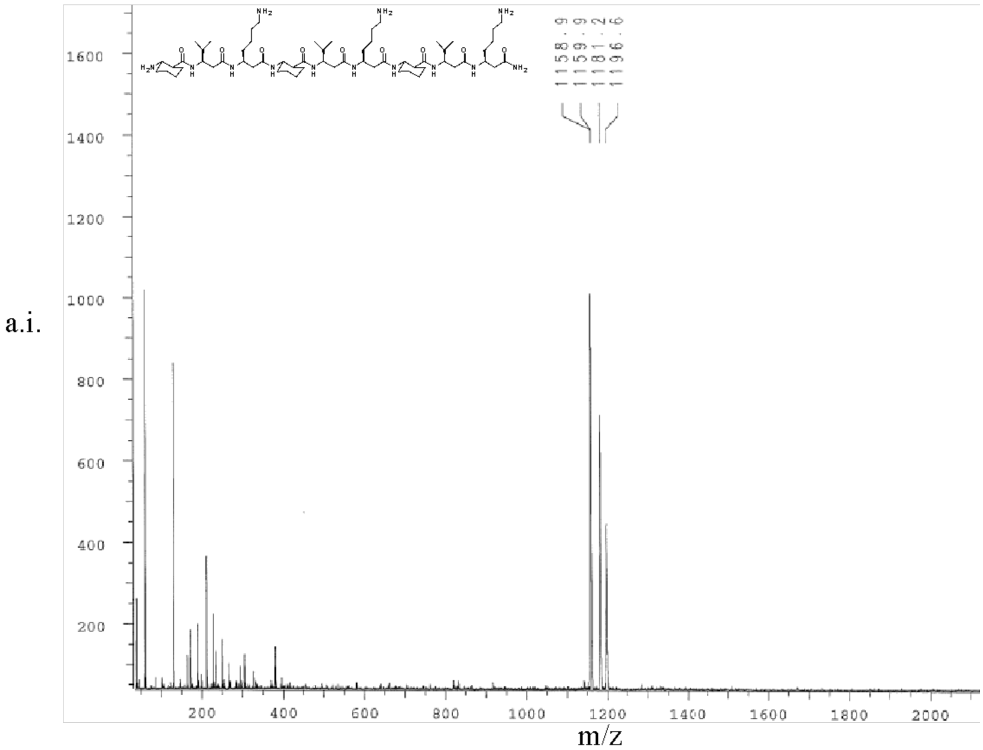

Figure A in S1 File. MALDI-TOF-MS Calculated for NH_2-(ACHC-*β*[3]hVal-*β*[3]hLys)$_3$, (#4). Expected mass for $C_{60}H_{111}N_{13}O_9$, $[M+H]^+$: 1158.9. Peak found at 1158.9.

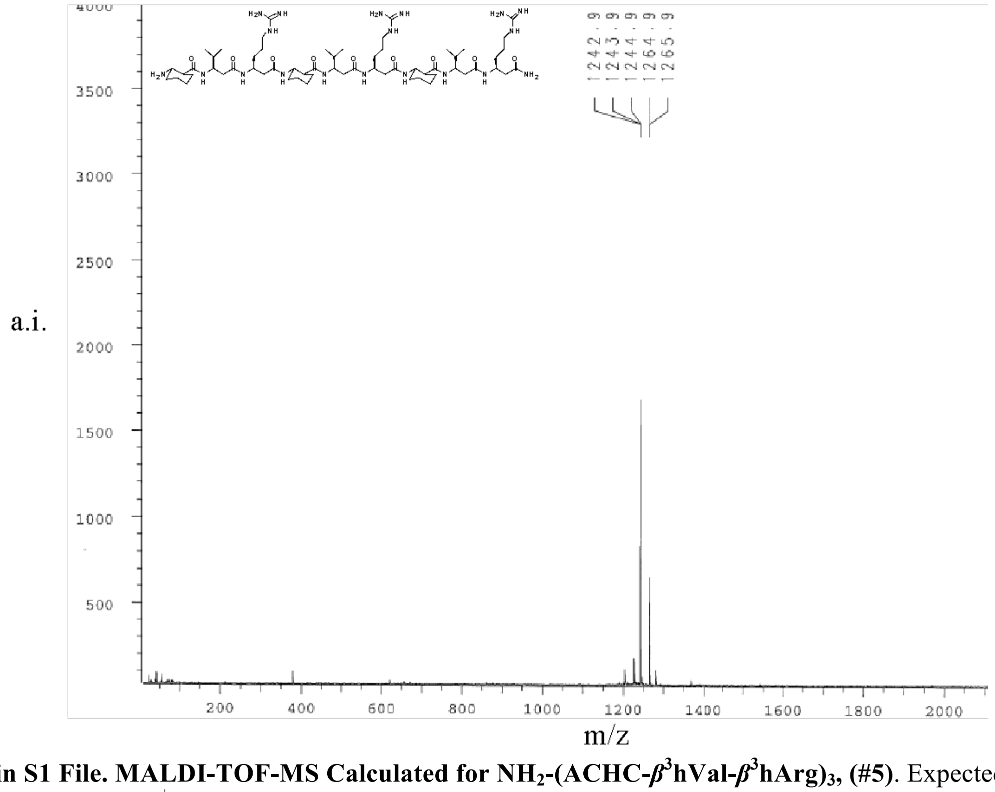

Figure B in S1 File. MALDI-TOF-MS Calculated for NH_2-(ACHC-*β*[3]hVal-*β*[3]hArg)$_3$, (#5). Expected mass for $C_{60}H_{111}N_{19}O_9$, $[M+H]^+$: 1242.9. Peak found at 1242.9.

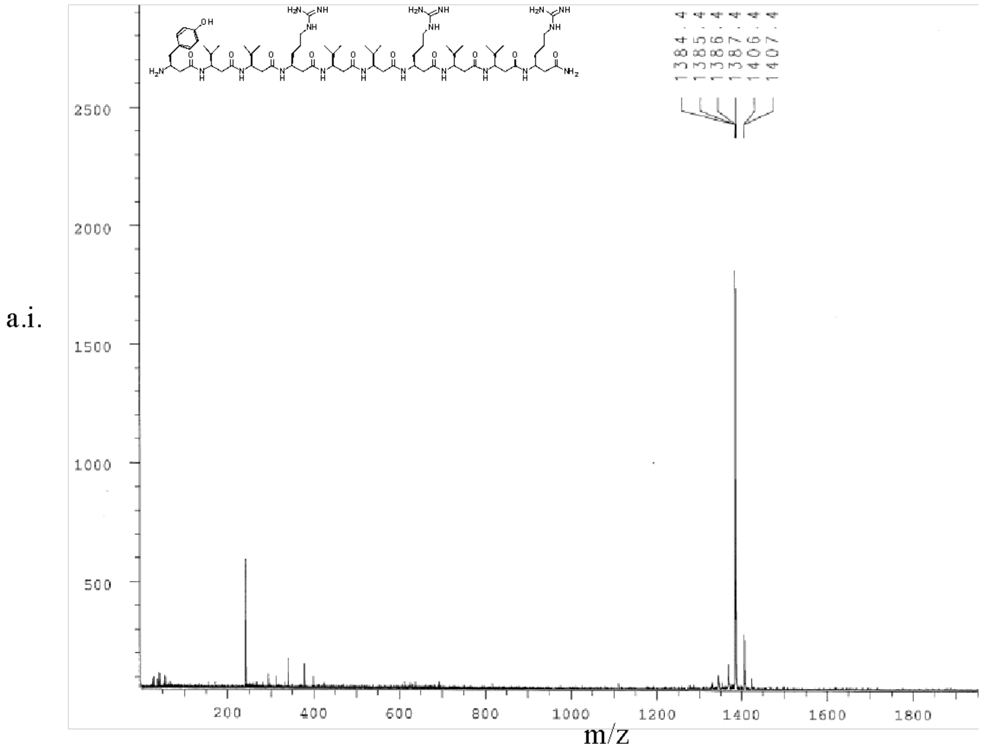

Figure C in S1 File. MALDI-TOF-MS Calculated for NH_2-β^3hTyr-(β^3hVal-β^3hVal-β^3hArg)$_3$, (#21).
Expected mass for $C_{67}H_{122}N_{20}O_{11}$ $[M+H]^+$:1384.0. Peak found at 1384.4.

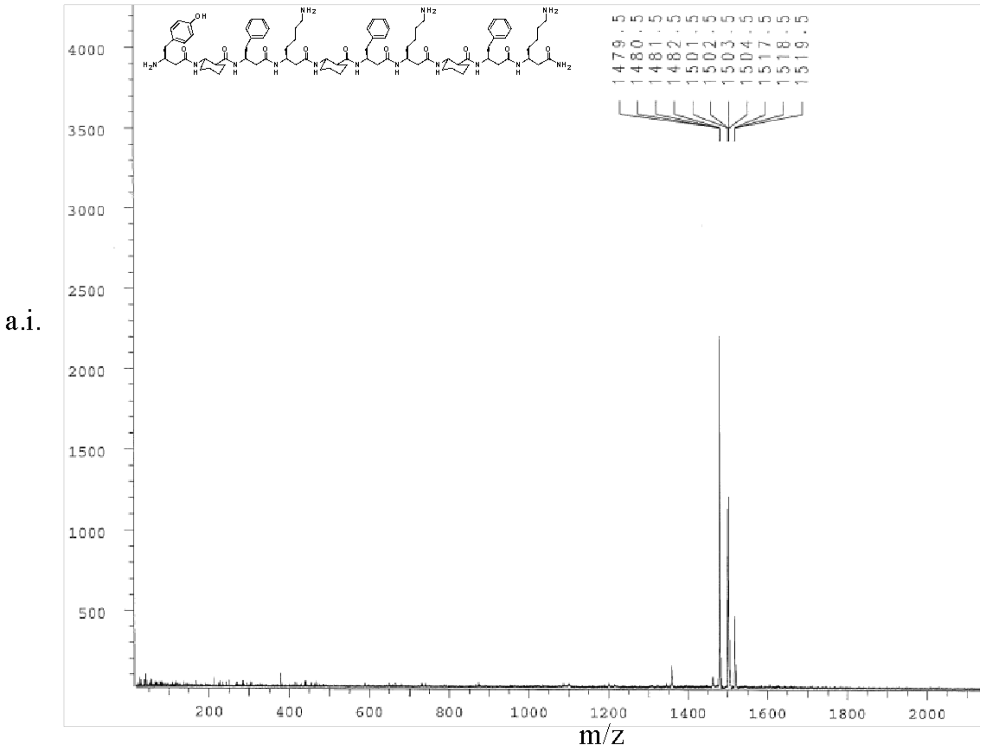

Figure D in S1 File. MALDI-TOF-MS calculated for NH_2-β^3hTyr-(ACHC-β^3hPhe-β^3hLys)$_3$, (#16).
Expected mass for $C_{82}H_{122}N_{14}O_{11}$ $[M+H]^+$: 1480.0. Peak found at 1479.5.

S 1.2 Dose effect Curves

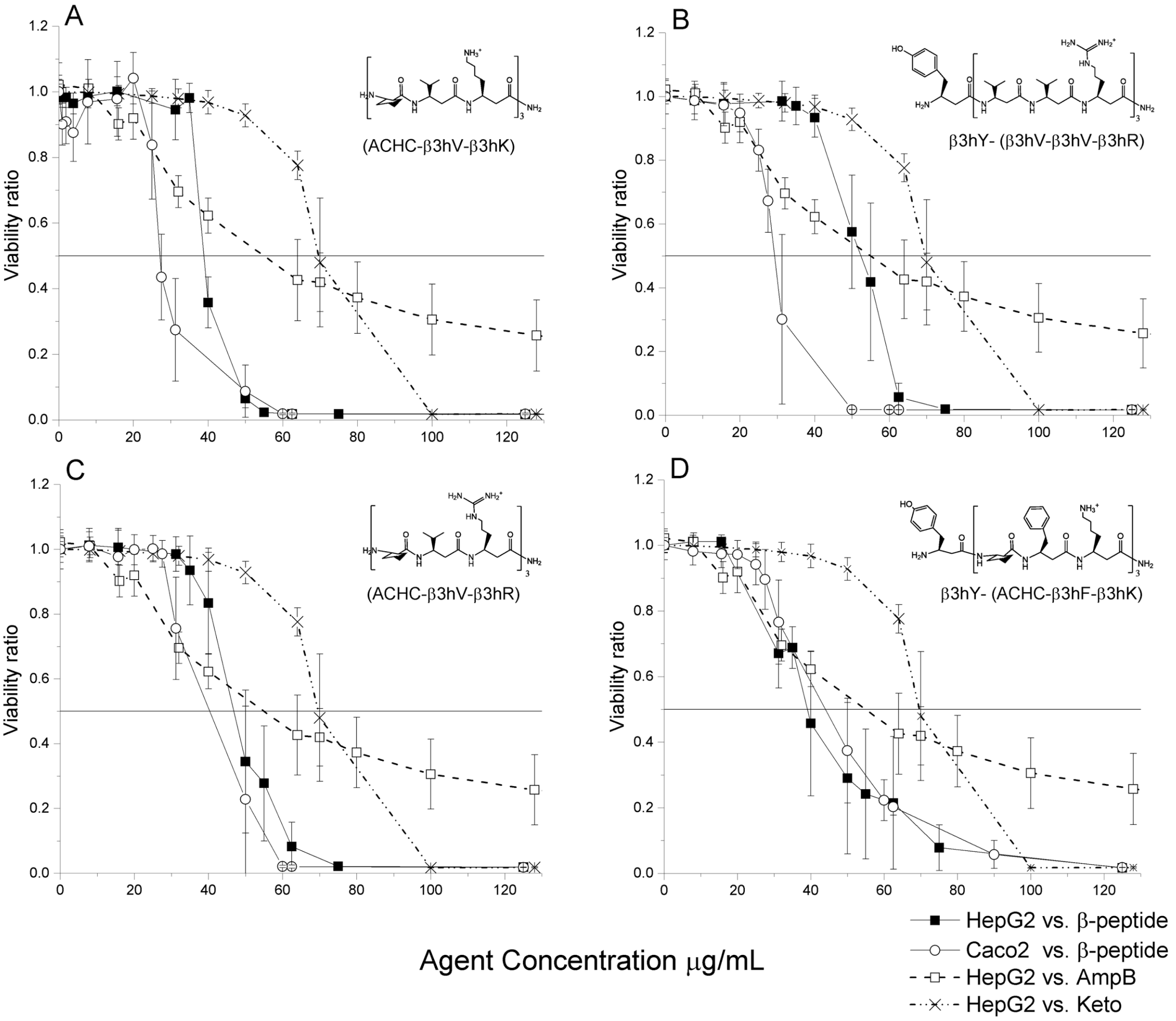

Figure E in S1 File. Dose effect curves for HepG2 (▪) and Caco-2 (O), exposed to a battery of β-peptides during 24 hours, correlated to dose effect curves for (▫) Amphotericin B and (x) Ketoconazole. The graphs were arranged based on the hydrophobicity of the β-peptide, which increases alphabetically. This is the mean of two independent experiments, each with four biological replicates. The error bars correspond to the standard error of the mean, n=8.

S 1.3. Clonogenic assessment.

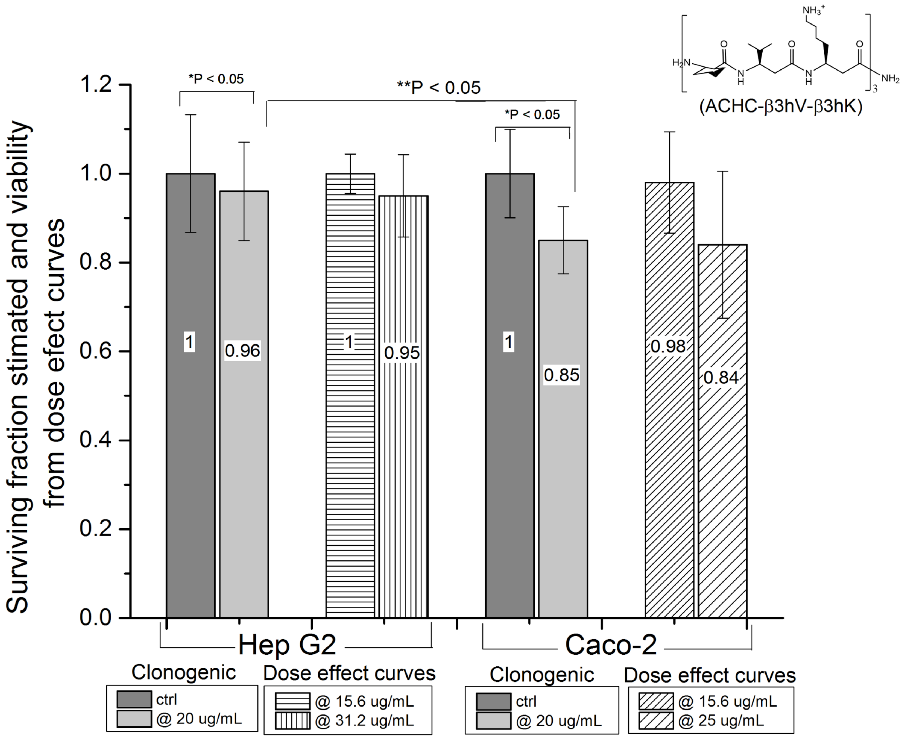

Figure F in S1 File. Clonogenic capacities of HepG2 and Caco-2 after (ACHC-β3hV-β3hK) exposure. Treatment underwent for 24 hours at 20μg/mL β-peptide. The assay was performed two independent times, each with three biological replicates. The plating efficiency was > 9.5% for HepG2 and > 22% for Caco-2 for treated cells. *Statistic analysis used was described by Gupta et al [53].** 2-Sample t-Test was used to establish that the means are statistically different.

S 1.4 Cell density-dependent toxicity assays. The clonogenic assays, used to evaluate the long-term effect of β-peptide exposure in both cell lines, were performed using a cell density proportional to the used for the cytotoxicity assay and the treatment was performed prior to seeding the cells in the six-well plate. HepG2 tends to form colonies, a feature which could affect the response to the β-peptide when compared with the monolayer growth of Caco-2. In order to evaluate the effect of the cell density upon exposure to the β-peptide, cells were seeded in a six-well plate (Fisher Scientific, GA, USA) and before the β-peptide treatment. Caco-2 and HepG2, 500 and 750 cells respectively, were seeded; this quantity was set to treat isolated cells without a colony formation signal. After seeding, they were allowed to adhere to the plates for 48 hours in DMEM. Then, cells were exposed to 16 μg/mL of the β-peptide model (ACHC-b3hV-b3hK) and the plate was allowed to incubate for 24 hours. After 24

hours, the plate was washed using HBSS, softly, to prevent dragging the cells. Once washed, DMEM was added to each plate and left for a period of 12 days for HepG2 and 10 days for Caco-2 to begin the colony counting process. These assays indicated that when we treat a significantly lower quantity of cells with β-peptide, the viability profile is affected. In this experiment, the cells were treated with a non-cytotoxic concentration of our model β-peptide, as determined in previous assays, 16 μg/mL. However, a significant level of cytotoxicity was obtained; approximately 25% for Caco-2, and an unexpected 55% was obtained for HepG2. This response could be explained if the individual growth morphologies are considered. In both instances (high and low density), Caco-2 grew in a monolayer. HepG2 cells form small aggregated colonies as shown in the confocal microscopy section of the main paper. The initial viability assessments were conducted in a 96-well plate, and 14,000 cells were seeded in each well, allowing them to overlap and form aggregates as was expected. However, when 750 cells were seeded in a six-well plate, instead of 14,000 cells, they seemed to spread out and did not overlap or aggregate. This growth morphology shift could render cells more susceptible as the surface to volume ratio is increased, thus exposing more cells to the β-peptide.

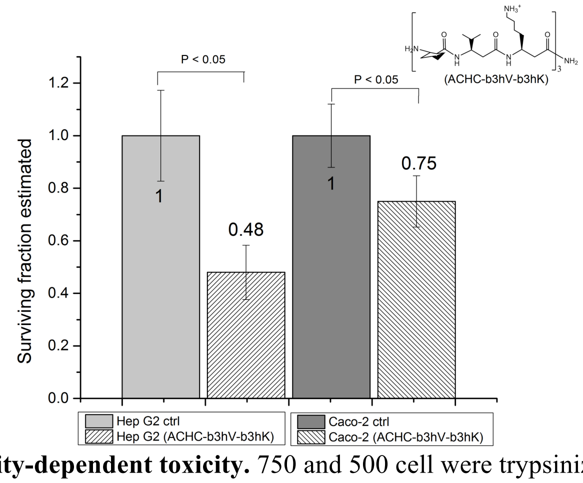

S1 Fig G. Figure Cell density-dependent toxicity. 750 and 500 cell were trypsinized and seeded per well respectively. After 48 hours, both were exposed to 16 μg/mL of (ACHC-b3hV-b3hK) for 24 hours. Cell viability was determined by CFU counting. The lowest plating efficiency obtained for HepG2 was 4% and the lowest for Caco2 was 10%. The statistical analysis was performed as described by Gupta et al [5].

References

[1] M.-R. Lee, N. Raman, S. H. Gellman, D. M. Lynn, and S. P. Palecek, “Hydrophobicity and helicity regulate the antifungal activity of 14-helical β-peptides.,” *ACS Chem. Biol.*, vol. 9, no. 7, pp. 1613–21, Jul. 2014.

[2] N. Raman, M.-R. Lee, S. P. Palecek, and D. M. Lynn, “Polymer multilayers loaded with antifungal β-peptides kill planktonic Candida albicans and reduce formation of fungal biofilms on the surfaces of flexible catheter tubes.,” *J. Control. Release*, vol. 191, pp. 54–62, Oct. 2014.

[3] S. H. G. A. Amy J. Karlsson, William C. Pomerantz, Keane J. Neilsen and S. P. Palecek, “Effect of Sequence and Structural Properties on 14-Helical b-Peptide Activity against Candida albicans Planktonic Cells and Biofilms,” *JACS Chem. Biol.*, vol. 4, no. 7, pp. 567–579, 2009.

[4] J. K. Murray and S. H. Gellman, “Microwave-assisted parallel synthesis of a 14-helical beta-peptide library,” *J. Comb. Chem.*, vol. 8, no. 1, pp. 58–65, 2006.

[5] N. Gupta, K. Lamborn, and D. F. Deen, “COMMUNICATION A Statistical Approach for Analyzing Clonogenic Survival Data,” *Radiat. Reserch Soc.*, vol. 145, no. 5, pp. 636–640, 1996.
